# Technology-Based Social Innovation: Smart City Inclusive System for Hearing Impairment and Visual Disability Citizens

**DOI:** 10.3390/s22030848

**Published:** 2022-01-22

**Authors:** Ignacio Chang, Juan Castillo, Hector Montes

**Affiliations:** 1 Facultad de Ingeniería Eléctrica, Universidad Tecnológica de Panamá, El Dorado, Panama City 0819-07289, Panama; ignacio.chang@utp.ac.pa; 2 Centro de Investigación, Desarrollo e Innovación en Tecnologías de la Información y las Comunicaciones (CIDITIC), Universidad Tecnológica de Panamá, El Dorado, Panama City 0819-07289, Panama; juan.castillo21@utp.ac.pa; 3 Centro Internacional de Desarrollo Tecnológico y Software Libre (CIDETyS AIP), Universidad Tecnológica de Panamá, El Dorado, Panama City 0819-07289, Panama; 4 Centro de Investigación e Innovación Eléctrica, Mecánica y de la Industria (CINEMI), Universidad Tecnológica de Panamá, El Dorado, Panama City 0819-07289, Panama; 5 Centre for Automation and Robotics CSIC-UPM, Arganda del Rey, 28500 Madrid, Spain

**Keywords:** technological integration, social innovation, early warning, risk management panel

## Abstract

The multilayer technology integration of hardware and software will reduce the social inclusion gap and increase the support in case of an emergency for people with special needs at hearing and visual levels. This research shows a development based on Internet of Things to support people with visual disabilities (PwVD) for indoor and outdoor activities. The decision-making process is made at the operational, tactical, and strategic level, providing a safe place so people with visual and hearing special needs can make decisions, their families can make decisions, and the government authorities can make decisions in case of an emergency or even on a day-by-day basis. In the case of the authorities, the smart visualization of the data according to the information provided facilitates Comprehensive Disaster Risk Management (CDRM) and Disaster Risk Reduction (DRR). The main findings are based on the need to develop mobile applications, dashboard and web applications that are responsive to people with visual or hearing disabilities, and the need to develop an infrastructure of communication systems assisted by batteries and clean energy, and independent of the current telecommunications system, to allow greater reliability.

## 1. Introduction

Technology is not an end, but a means or a tool in favor of human needs, especially in conditions of disability, for Latin America. This is due to the lack of social balance faced by people with disabilities and the absence of technological means and solutions, in these countries, that allow these people to develop individually and collectively. According to the World Bank, 15% of the world population [1], about 1 billion inhabitants, have some type of disability. Therefore, from a particular point of view, which is related to the topic discussed in this article, it is evident that people with disabilities are more vulnerable to emergency situations than people without disabilities, and even to the challenges they may face, in their day-to-day life, within their closest environment [2].

Therefore, technology has played a preponderant role in the inclusion of people with disabilities, including accessibility designs in different settings and environments [3,4,5], the developments of applications and hardware technological tools [6,7,8], and monitoring systems that track location [9,10,11], among others. Likewise, there are studies on technical considerations to be considered for technological developments [12,13].

In addition, in [13], the following is stated: “Position and time are some of the most important pieces of information for convenience, safety and security in our daily lives and have enabled groundbreaking advancements in many fields of science and engineering.” Based on this approach, some research questions arise: How can one obtain the position and time of people with disabilities? How can one use position and time in the underdevelopment of communities without sustainable infrastructure? What is the level of reliability of position and time identified by devices used in Internet of Things (IoT) systems?

The above questions are of vital importance to many people, including those with disabilities. Some solutions proposed by the scientific community are related to the use of IoT [14] in facilitating the assistance and integration of technologies to improve their quality of life, connectivity, and travel in cars [15,16], or the use of tech tools that can help with safer houses and cars [17,18].

In recent years, systems based on electronics, radio frequency, and smartphones have been designed in the Republic of Panama to assist in the mobility of people with visual disabilities (PwVD). These systems have been designed and implemented within the framework of two projects known by the acronyms MOVIDIS and MOVIDIS-II, which have been developed to provide mobility aids for people with visual disabilities in public passenger transportation and, also, in building interior environments [19,20,21,22,23,24,25,26]. Another project has also been carried out in which an early warning system, based on electronic and telecommunications technology, has been designed to assist people with visual or hearing disabilities [27].

Given the high volume of people with disabilities in the world and the continuous advancement in the age of people, it is easy to predict that accidents and diseases are gradually going to affect many people in the world. Therefore, the following question arises: What actions must be undertaken to achieve full and effective participation in society on equal terms with others? The answer lies in the search for solutions that have been developed in different research groups, in public and private institutions or organizations, and in the social policy programs of many countries.

The objective of this work is to integrate the developments of three projects, conducted by our research group, to implement an inclusive early warning and disaster risk reduction system, with new features, to provide rapid and early responses to disaster events, particularly, for flood risks in the hydrographic basins selected for this study. For this purpose, we have examined the literature review presented in the introduction, considering that there are differences between the different geographical areas and the types of disaster risk studied.

In the literature, there are several systems, tools, and computer platforms designed to collect information relevant to risk management and disaster risk reduction. For example, (i) the Copernicus Programme of the European Union for Earth Observation [28]; (ii) the research program in integrated risk management and adaptation to climate change of the Corporación Universidad de la Costa, for risk reduction of flash flood hazard and atmospheric phenomena in the Metropolitan area of Barranquilla, which allows authorities and community, in general, to make decisions for disaster prevention [29]; and (iii) a study on safety and health at work in Colombia, as challenges for people with disabilities [30].

Other studies show that most of the early warning systems (EWS), designed for watersheds, focus mainly on the technological characteristics of the system, finding interesting innovative solutions, considering some aspects in human factors [31,32]. However, they have not considered the concept of social inclusion for people with some kind of disability, as it is presented in this manuscript. From this point of view, improving the infrastructures of the proposed systems in [31,32] is required, increasing the level of awareness of the population regarding their reaction to risk situations, but also in providing aids to people with disabilities. The United Nations International Strategy for Disaster Reduction (UNISDR) recommends the establishment of national people-centered EWS [32]. However, it does not indicate how this type of EWS should be built and the steps needed to implement it. Although there are general strategies for the creation of EWS, it is understood that in each country, new proposals must be developed for them to be adapted in their regions, as well as implemented and put into operation. It is evident that this system will be more complex when, in addition to being people-centered, people with disabilities are also considered. The proposal presented in this document considers an EWS and a disaster risk reduction (DRR) system that considers people with visual impairment and people with hearing impairment, which is interesting.

There are several EWS projects in Latin America and the Caribbean, where some are in operation at a medium scale, and others are under development, and where data collection is performed by means of radiofrequency systems, microwave links, or fiber optics-based systems [33]. These data are evaluated at the reception centers by technical equipment and processed through general statistical models. Until 2016, most EWS in Latin America were not managed in real-time, nor did they include people with disabilities [33]. By 2018, in Ecuador, an EWS with certain inclusive features was designed for risk reduction in the case of Cotopaxi volcano eruption [34], however, it is not yet operational. In most EWS, both monitoring and early warning service, dissemination, communication, and response capability are not fully inclusive. This is stated in [35], where a study of disability inclusion in an early warning system in flood prone areas in Bangladesh was conducted. However, several studies highlight the importance of incorporating people with disabilities in information and communication in early warning systems, in various events, for the reduction of risks that may happen [32,35,36].

In order to have a better operation of the EWS, in the technological design, its instruments, and its management and operation, a community-based disaster risk reduction system should be considered, which also involves expert institutions in this type of tasks, and appropriate state policies [37]. In this case, the community residing in the area subject to risk should be trained for its understanding, care, and relevance, with the aim of their participation in specific tasks of risk monitoring and issuing warnings at the given time. In this way, the early warning system can be extended to other areas as a source of a research, education, training, and emergency response ecosystem, as a mechanism to empower the community and enhance its resilience [38]. These concepts, adapted to flood risks, are also within the proposal presented in this article.

An important aspect to be considered is that EWSs should be of open data, real-time, and with the ability to create a multifunctional data dashboard [39] useful for the community, authorities, and the public and private sectors. In [39], a real-time data dashboard was designed to manage data from cyclone arrivals. In our proposal, this concept is considered for the creation of a monitoring dashboard for flood risk in hydrographic basins.

In addition to the above, the United Nations Development Programme (UNDP) provided in 2018 five important aspects for the functional design of early warning systems [40], which are based on: (i) Institutions, regulations, and capacity development, (ii) Technological solutions, (iii) Community outreach and community-based solutions, (iv) Private sector engagement, and (v) International Co-operation and Data-sharing. It is assumed that the implementation of the EWS considers these aspects, however, in each area where they are being designed and implemented, they have particular characteristics that depend on the type of disasters to be addressed, the social and technological infrastructures, and the community itself.

With regard to Latin America, ECLAC (Economic Commission for Latin America and the Caribbean) created the Regional Observatory for Development Planning as a dynamic space for analysis, information, and collective knowledge building for governments, academia, the private sector, and civil society on development planning in Latin America and the Caribbean [41]. This instrument will enable anticipation and better preparedness for natural hazards. In this regard, it can be said that the proposal presented in this article will contribute to the expansion of this Regional Observatory and may serve as a guide for other related projects in the region. As can be noted in the above description, the trend is the creation of early warning systems as community-based disaster risk reduction systems, as a participatory ecosystem. Additionally, as an important aspect, it is very important that the new EWS systems are inclusive. Such is the case described in the new proposal presented in this manuscript.

A number of natural disasters have highlighted the need for effective disaster risk reduction, as many of them are unforeseen. Nevertheless, further work should be conducted on improving DRR, considering multiple UN policy frameworks, primarily because it is a significant component in mitigating the increasing environmental and socioeconomic costs of disasters globally [42]. This requires specialized DRR guidelines for regions with biodiversity and multiple hazards such as mountains and river basins, etc., emphasizing conservation, restoration, and sustainable ecosystem management as key elements [42], and also taking into consideration the five approaches to building functional early warning systems [40].

In the community framework, it is possible to consider EWS within the Smart City concept as a large and permanent human ecosystem that provides a set of services and opportunities to citizens [43,44] and where a multidimensional service classification is suggested along with the development of the required basic infrastructure. This involves the combination of Big Data and Internet of Things to enable the integration of different technologies and paradigms within a large smart city ecosystem [45], with its respective architecture and data dashboard [46], which also includes healthcare [47,48]. Additionally, it should be highlighted that the monitoring of elderly or disabled people has been given much importance [48], for their care and considering their informed consent, which leads to the determination of the needs of a smart city for sustainable development from the information system that is generated [49].

Considering health care management, the COVID-19 pandemic demonstrated many weaknesses in hospital medical systems around the world, exposing the lack of care plans for people with disabilities or the most vulnerable [50]. In other words, there was a general lack of preparedness to include these people. This was because their needs were not considered in urban health policy, planning, and practice [51], and this is even more so in the rural sector. What can be achieved to reduce the negative effects of these weaknesses? As is well known, these facts generated distrust in many sectors of society. However, some designs can be found with functional strategies for health management and disaster resilience [52]. However, as mentioned above, each region has its own particularities, so that new designs for health care management, among other details, must be adapted.

As can be noted, in the literature review described above, no automated systems were found for monitoring, control and decision-making, integrating early warning systems, information systems that monitor disaster risk levels, systems for health care management in hospitals, or for autonomous movement for people with visual or hearing impairment in enclosed spaces and in public passenger transport, except for some combinations, as in [53]. In that instance, they combined EWS and IoT for the development of sensor-based wide area networks to measure levels of solid particles in the air and determine air pollution. In addition, it is suggested that designers of digital services for citizens consider the diversity of the target audience and their particular needs in a disaster situation. The concept of Universal Design [54] should be considered as much as possible, always including people with disabilities and people with special needs in technological designs.

This article is structured as follows: Section 2, Section 3 and Section 4 present a brief description of the systems designed by the research group of this proposal, which will be integrated for the creation of a new inclusive early warning and disaster risk reduction system, and which could serve as a model for other developments in other regions. Specifically, Section 2 presents the description of the design of an inclusive early warning system to incorporate visually and hearing-impaired people into the current EWS. Section 3 describes the systems developed for the mobility of visually impaired people in public passenger transport and inside buildings. Section 4 presents an information system with a monitoring dashboard that categorizes disaster risk, presents relevant data for decision-makers, and contributes to disaster risk reduction in each area. Finally, Section 5 and Section 6 present a discussion of the potential offered by the fusion of the three designed systems and conclusions, respectively.

## 2. Inclusive Early Warning System 

The accelerated growth of the population, which increases the demand for energy, food, and environmentally unsustainable activities, affects us all through what is called climate change, the effects of which are observed increasingly frequently, as intense and destructive floods, droughts, and storms, generating emergency situations or greater risk of disaster. In addition, the latest socioeconomic crisis has been aggravated by the COVID-19 pandemic, creating new realities, challenges, and opportunities around the world.

**Figure 1:**
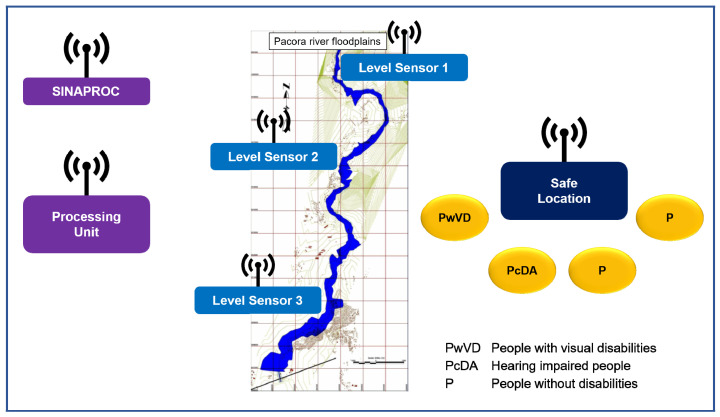
The conceptual model of the inclusive early alerting system design [27].

Based on a civil society collaboration and the research team of the Universidad Tecnológica de Panamá (UTP), a first inclusive early warning system was design to support blind and hard-of-hearing people [55,56,57]. The implementation of this system and its validation has positively influenced society. This system, of modular type, can be replicated to other groups with similar needs. Figure 1 shows the conceptual design of the monitoring, warning, and disaster risk reduction system.

As can be seen, it has three level of data capture points, one in the upper basin, another in the middle, and the last in the lower one. The data collected are transmitted to the processing center that is responsible for generating the alert when it is detected. This alert is sent to the Civil Protection System (SINAPROC) of Panama, which distributes it in the affected basin. The local authority and civilian emergency support organizations, such as the Red Cross, receive the alert signal issued with information on the safe sites, the number of vulnerable people in the emergency area, and their location.

Likewise, it is observed in Figure 1 that, in the event of a flood or emergency, there is a “safe place” as a refuge. When the alert signal reaches it, a warning is generated for all remotely connected people in nearby vulnerable areas, telling them to leave the area if necessary. In the case of the Pacora river, where the study was carried out, the hydraulic model of the river gave as a result the time for eviction as 45 min, which is enough time to leave the place. In the case of PwVD or people with hearing disabilities, the system assisted them to move autonomously to the nearest safe place.

It is planned that the system model will be installed in 10 priority basins in the county, while the warning mechanism or tool should also protect the people who travel through these basins.

Likewise, it is considered an induction program for the community, as well as documentary material for the schools in the areas, and evacuation programs in the event of a flood emergency.

It has had the support of the Central American Higher University Council (CSUCA) and the Swiss Agency for Development and Cooperation (SDC), as well as the support of USAID’s Bureau for Humanitarian Assistance (BHA) of the Regional Disaster Assistance (RDAP) program, the Red Cross, the Association of Municipalities of Panama (AMUPA), the Assistive Innovation Office of the National Disability Secretariat (SENADIS) of Panama, the National Union of the Blind of Panama, and local authorities in the analysis and development of the prototype of the inclusive early warning system described.

In this way, a very important information system is created for decision makers and the community in general. At the same time, it is satisfactory for improving the quality of life of people in the beneficiary populations, including people with visual or hearing disabilities.

**Figure 2:**
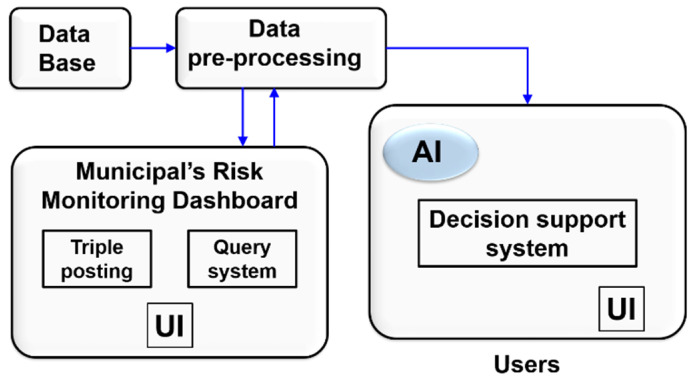
Merged Model.

It is also recommended to merge the designed real-time early warning system (see Figure 2), comprising the 10 priority hydrographic basins chosen, with the risk monitoring dashboard created by the Association of Municipalities of Panama (see Section 4).

On the other hand, the following aspects should be considered:

- For a successful implementation, effective coordination with local authorities, leaders, and residents is required.
- The design is adapted to the conditions of the basin to be installed and has a multidisciplinary team with defined responsibilities and who are motivated towards its execution.
- The UTP will initially provide support from a Research Center with instruments and resources.
- This design meets Sustainable Development Goals SDG 5 (gender equality), SDG 10 (reduction of inequalities), SDG 11 (sustainable cities and communities), and SDG 17 (partnerships to achieve the goals).

**Figure 3:**
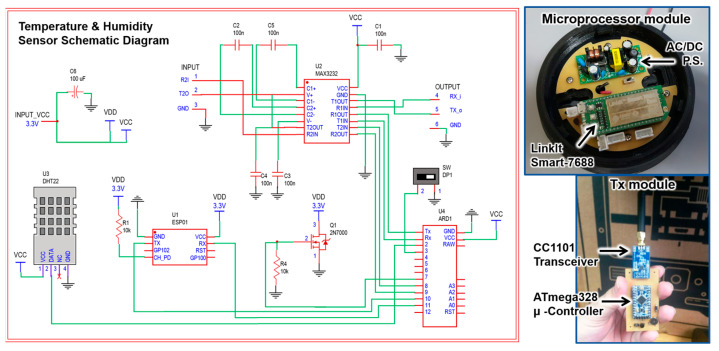
Design, electronic components, and model blueprint [57].

Figure 3 shows partial views of some components, electronic designs, and maps generated during the study. Finally, this system allows the automation of tasks quickly and easily. Human intervention in their development decreases, reducing the risk of human errors during execution, which could become serious, because the main objective of an early warning system is to forecast the real threats to protect the life and property of the citizens.

## 3. Mobility of PwVD in Public Passenger Transport and Inside Buildings

In the Republic of Panama, people with visual disabilities amount to more than 83,000, according to the National Office of Comprehensive Health for the Population with Disabilities of the Ministry of Health of Panama. However, for these people, due to their visual limitations, it is not easy to reach a desired destination autonomously and they do not have the ease of being able to move around inside buildings.

Very few PwVDs, who can move within various environments, indicate that on many occasions they lose connectivity on their smartphones in closed spaces. For this reason, a team formed by several research groups from three regional headquarters of the Universidad Tecnológica de Panamá have developed two research projects to design different prototypes based on ICT, to facilitate mobility of people with visual disabilities in public passenger transport in Panama cities and in building interior environments.

The first of the projects is known by the acronym MOVIDIS (Mobility of People with Visual Disability) [58], which offers solutions to assist with mobilization of these people in public passenger transport, and the second has the acronym MOVIDIS-II [26], which offers solutions to help guide PwVD inside buildings.

### 3.1. Public Transportation Design

In the case of public passenger transport, two prototypes were designed, one based on the development of applications for smartphones and the other on the design and implementation of electronic modules that communicate by radio frequency within the ISM band (Industrial, Scientific, and Medical −433 MHz). The final objective is the use of redundant systems to always guarantee the connectivity of the PwVD, from their access of the bus at the initial stop to its destination. There is the option of monitoring this person by a close third person, considering the privacy and legal guardianship permissions.

The first system is made up of three smartphones, the one that uses the PwVDs, the one that is manipulated by the bus driver and the one that has the guardian of the PwVDs, considering the assigned legal guardianship permits. The second prototype consists of three electronic devices, one that carries the PwVDs, another installed in the bus, and another in the respective stops.

**Figure 4:**
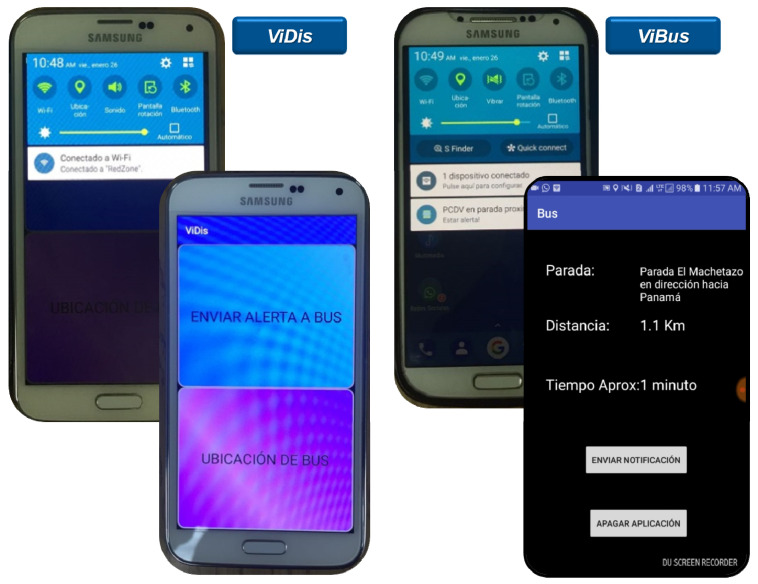
ViDis and ViBus mobile applications for Android OS [19,20,22].

Multiple experimental tests have been conducted with both systems developed, independently of each other, obtaining promising results. In the articles referenced above, you can see the main results achieved, and the project website (http://movidis.utp.ac.pa/movidis-i/, accessed on 22 October 2021) shows other evidence in video format, among others. Figure 4 shows the selection screen and the main screen of the ViDis application (on the phone that carries the PwVDs) and the ViBus application (on the phone that is on the bus) installed on the respective smartphones.

**Figure 5:**
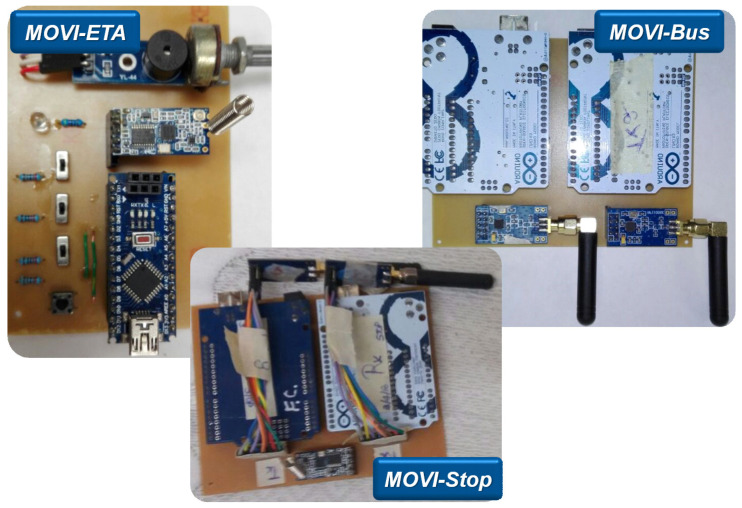
RF Prototypes developed for the MOVIDIS project: MOVI-ETA, MOVI-Bus, and MOVI-Stop [19,24].

For the second system, three dedicated electronic modules have been developed, which communicate with each other, within the designed protocol, in the RF ISM band. Figure 5 shows the first developments of these electronic modules, namely MOVI-ETA (module that carries the PwVD), MOVI-Bus (module installed on the bus), and MOVI-Stop (module installed at stops). The MOVI-ETA can communicate with the MOVI-Stop so that the PwVD can reach the stop and, with the MOVI-Bus relaying the information from the MOVI-Stop, request to board the bus.

**Figure 6:**
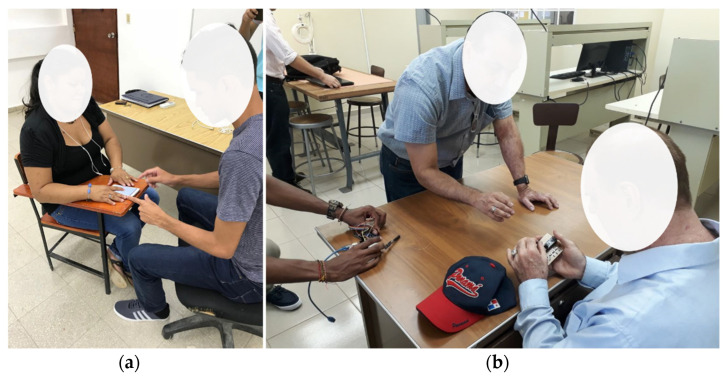
PwVD prototypes training. (**a**) Mobile training. (**b**) RF Device training.

The validation protocol of the developed systems begins with a preliminary training session for the people who are going to make use of the prototypes. Initially, people without visual impairment were trained to assess them in a first phase, then later, PwVD were trained, who collaborated with the project. It can be said that the training time in the use of developed applications and electronic devices is relatively short because their interfaces have been developed under the concept of user-friendly and intuitive. Figure 6 shows the training sessions of both prototypes with PwVD, volunteer collaborators of the project.

With the knowledge of the results of both systems described above, it is proposed to integrate them into a new expanded system to constitute a sensory monitoring network, where there is redundancy, to rule out some inherent communication errors depending on the environment and the communication infrastructure at the site where it would be used.

Although, initially, the main beneficiaries of these technological developments will be university students with visual disabilities, who will use the prototypes designed from their respective residences to the university, specifically, in well-defined and proven routes, these systems will be considered in extended use, i.e., as a universal technology. This is because, as mentioned above, many people will have the possibility to use them over time, as the systems can be integrated into the urban passenger transport system.

### 3.2. Designs for Moving around Inside Buildings

In this case, two ICT-based systems were also designed and implemented. In the first one, the PwVDs use the smartphone and communicate with the environment through devices based on Bluetooth Low Energy technology called iBeacons. These beacons contain the necessary information regarding the place where they are installed, such as their Cartesian position and the important details of the nearby places in their immediate vicinity. The interaction of the information contained in the beacons and the algorithms installed in the phone make it possible for the user to move within that environment. The second system consists of an electronic module that carries the PwVDs and communicates with the environment by means of the RFID (RF identification) tags installed on it. These RFID tags, such as the iBeacon devices, contain enough information for the PwVD to recognize the environment and move around by receiving audible instructions from the devices they carry, in both cases.

**Figure 7:**
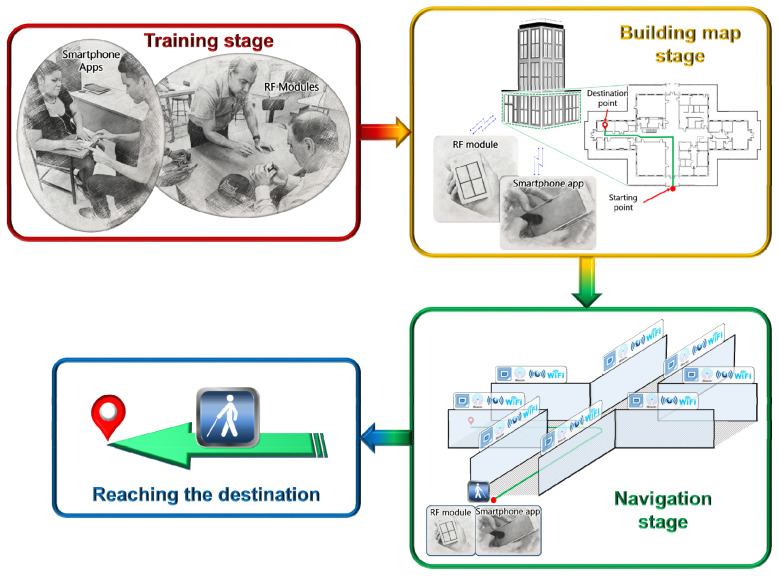
Conceptual Model for movement inside the buildings [10,12,25,26].

Multiple experimental tests have been conducted with both systems developed, obtaining good results. In the articles referenced above, you can see the main results achieved, and the project website (http://movidis.utp.ac.pa/proyecto-movidis-ii/, accessed on 22 October 2021) shows other evidence in video format, among others. In Figure 7, a conceptual diagram of the planning of the system is presented, divided into four different, very well-defined stages, and those are:

- Training stage. After the development of the proposed systems, each research group will train the PwVDs in the use of these systems and in the action protocol that they must carry out during their use.
- Stage of loading the building floor plan. When the PwVDs are going to access a building, specifically a floor of it, they can load the access plan to it in relation to the sensors and the database that are installed in it, although it is also contemplated that they carry the floor plan previously, downloaded from a project server. These plans may vary in detail depending on the complexity of the building. The project will consider building floor plans of institutions that cooperate with the project.
- Navigation stage. The PwVD will be able to navigate in the building but taking into account a specific route (at the same time) that they will be able to load at the beginning of their route, or before entering the building. Inside the plant, the systems that the PwVDs carry will interact with the sensors installed inside the building, which are mainly iBeacons devices and RFID tags.
- Arrival at the destination point. Finally, the PwVDs will reach the destination point previously established in the system they have used, the app, or the RF modules.

Considering the designs and developments of inclusive EWS and systems for PwVD mobility aids in public transport and indoor environments, it is considered suitable to merge them in order to provide new positive contributions to people with disabilities. For example, if any type of mishap occurs, where an eviction alarm is activated, it is necessary to mobilize people to a safe place, including people with disabilities. Therefore, the simultaneous use of the systems described in this article is necessary, so they must have a channel of communication or automatic interaction between them. In this way, the PwVD can be moved to a safe area, and, at the same time, their position could be known in real-time, within the corresponding legal requirements.

## 4. Dashboard and Data Driven Decision-Making

In 2020, a risk monitoring dashboard was developed between the Universidad Tecnológica de Panamá and the International Center for Technological Development and Free Software AIP, with the support of the USAID/BHA RDAP program in service of the Association of Municipalities of Panama, as a pilot plan for the implementation of risk management tools aimed at a particular municipality, but with the capacity for growth.

The model developed was based on an adaptation of a disaster risk matrix, translated into an online form that uses historical data that are then presented in a dashboard that allows visual monitoring in real-time of the most relevant information generated by the government activities. In this way, decision-making is supported based on each strategy established by the authorities, based on the smart visualization of data in accordance with municipal information, offering a specific view of the information for all the actors involved in the Comprehensive Disaster Risk Management (CDRM) and, in turn, each of them can provide information, which constitutes an important step towards an efficient and citizen-centered organization.

**Figure 8:**
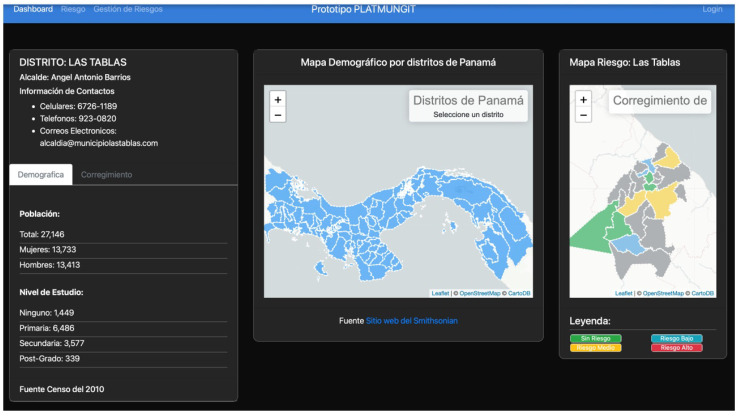
Risk monitoring dashboard prototype.

Figure 8 shows an image of the prototype risk monitoring dashboard designed that offers an interface with different graphic components such as a risk map, graphs, or demographic information panels for this information to support decision-making in matters of risk prevention. It should be noted that the control panel can be consulted by the public.

The characteristics of this panel are the following:

- Facilitates data entry to the information system designed;
- Friendly and intuitive, making it easy to update;
- Facilitates their growth;
- An information system accessible to citizens;
- A site with data that allow R + D + i activities and that always know the situation.

**Figure 9:**
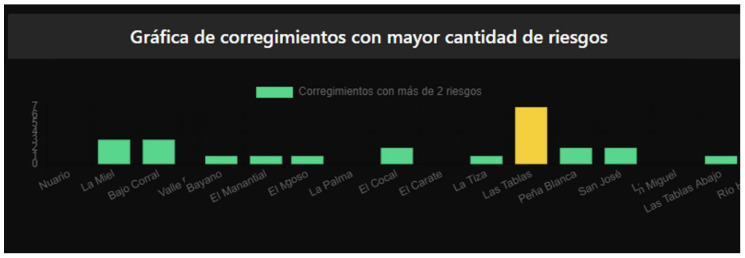
Bar Graph based on municipalities with their risk levels.

As can be seen in the lower part of Figure 8, a bar graph is shown where the districts with the highest number of risks are described. The bars change color according to the amount of irrigation, where the green color represents the districts that have less than 5 risks, yellow for those that contain more than 5 but less than 10, and red for those that are above 10 risks (See Figure 9).

These types of tools also have an impact on citizens with disabilities because they make it easier for municipalities, in case of emergencies or disasters, to consider them in risk management and humanitarian response to disasters. The name of the model and of this risk management dashboard is PLATMUNGIT (http://siglosrrd.org:3001/, accessed on 18 January 2022).

This project strengthens the government sector, in this case community or district, in risk management using information and communication technology to facilitate decision-making using a risk indicator by district.

## 5. Model Discussion

In this article, three prototypes of systems have been presented that have as an innovative aspect the inclusion of people with visual and hearing disabilities to assist them in the different areas described. Each one presents its main characteristics, the beneficiaries, the impact on the community, its limitations, and its ability to scale, which represents a great potential for use and assistance for people with disabilities, among others, if they were integrated or merged. However, this aspect of integration of the systems presented in this article has a high potential for feasibility and is achievable, because some research groups have participated simultaneously in the development of the projects described. Therefore, there are research groups that know the design, implementation, and operation of the three systems presented, making it possible to conduct a larger project for their integration.

**Figure 10:**
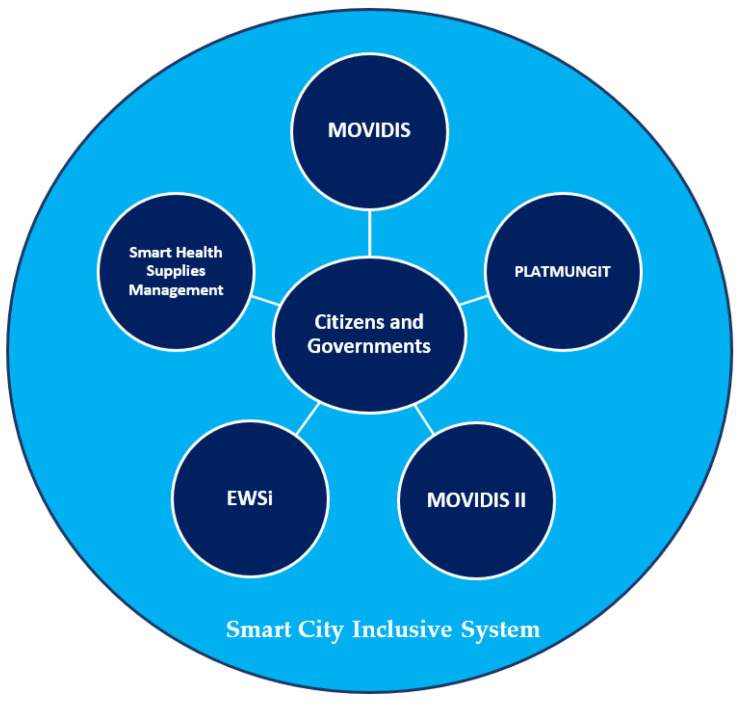
Smart City Inclusion System based on RRD System Modelling.

In this way, taking into consideration the advantages and characteristics of the existing designed systems, a new disaster risk reduction (DRR) system is proposed that merges the EWS (which will be expanded up to 10 river basins), a mobility system in public transport of passengers and indoor environments in buildings (which has a pilot plan for implementation and validation in a specific area of Panama City) and, finally, a risk monitoring dashboard that will be implemented as a pilot plan in a province of Panama. This approach includes people with visual and hearing disabilities, as well as people who live in monitored risk areas. Figure 10 shows the main components of the proposed system.

### 5.1. Support System Designed for Inclusion

The new system, which includes people with visual and hearing disabilities, will issue several types of early warnings:

- Before an imminent head of water or flood. In this case, it covers people who are in the areas vulnerable to flooding in the hydrographic basins that have the system installed.
- In an emergency occurring in public transport or in indoor building environments. Upon receiving this signal, the subscribers, if they are PwVD or people with hearing disabilities, will receive the action protocol on the device they carry so that they can evacuate and move to the nearest safe area. Simultaneously, the legal guardian of these people will receive a notification on their mobile phone, indicating the route, code, and address where the bus is located, or the address and name of the building.
- Visualization of risk areas and data of the types of risk are contained in the risk monitoring dashboard of the municipality of the subscriber. The person will be able to evaluate at any time the existing risk levels, considering the most appropriate actions according to the case. In the case of PwVD or people with a hearing disability, they will listen or read the risk level of the area where they want to be or visit.

The risk management dashboard would graphically show the elevation level of the rivers incorporated into the system. It is foreseen that because the inclusive EWS (EWSi) will be installed in 10 priority hydrographic points in the country, the subscriber will always be able to access real-time information on the rivers’ levels. Because the dynamic model of the river is available in real-time, if the subscriber is in a vulnerable area, at a time of risk, this person will receive an alert at the appropriate time to leave the area, thus reducing risk.

In addition, the information system provided by EWSi will be very useful for the national body that requires it, as this system can be used to capture other types of data and transmit them. Thus, there could be an information medium for other state or private entities.

Regarding the sensory network that will be formed in the public passenger transport system, its use will benefit not only the passengers of the routes established in the country but also national or foreign visitors who make use of the proposed system. In a similar way, the same thing will happen in indoor environments of buildings. However, the risk monitoring dashboard will only have the risk level of the area where the bus circulates. In any case, the number of people on the bus at the time of the accident or emergency will be recorded, which can only be known by the corresponding authorities.

This system will be available in a version for smartphones and in a portable electronic device, mainly for people with visual or hearing disabilities.

The risk managers of each district and municipality will be responsible for keeping the pertinent information updated, and the community in general will be able to send them information on important events that generate some type of risk for the community.

### 5.2. Design and Implementation Phases

For the integration of the designed systems, creating a sensory network based on the Internet of Things (IoT) is proposed, which will help to create, administer, and manage communities. It will also include artificial intelligence and data mining techniques.

A community participation phase is included in the final design (co-creation) and in the implementation of the system to achieve the empowerment of potential users of the system. This stage includes an induction on comprehensive disaster risk management and DRR. If possible, together, they will create the community risk plan.

The need to develop an infrastructure of communication systems assisted by batteries and clean energy independent of the current telecommunications system is also contemplated, to allow for greater reliability. Likewise, if there is no connectivity in the coverage area, there will be an alternative of wireless nodes for transmission and reception of data and alarms, to a point where this problem disappears.

Another aspect to consider is that each system to be integrated has redundancy. For example, in the case of inclusive EWS, an observability matrix is suggested with the historical data of each sensor so that, if any aspect fails, it is activated and the system continues to function while the fault detected is notified to the person in charge for its repair; but other redundancy alternatives can be evaluated.

In the event of a pandemic or an epidemic, considering the privacy and legal guardianship permissions, the Ministry of Health (MINSA) will have access to information on how many people with visual and hearing disabilities are in the system, to evaluate the measures to be taken if necessary. An important challenge is the integration of MINSA information with information from the Ministry of Social Development (MIDES) and municipal and district information systems. The challenge of this proposal is there, because community assistance is decentralized to the townships, who coordinate all social and health assistance in emergency situations.

In addition, in the dashboard of risks by township, the number of people with some disease (registered in the health services), vaccinated or cured must be made visible. Likewise, MINSA will evaluate the pertinent or related information handled by this panel to reduce misinformation based on outdated and false data.

On the other hand, it is important to study the key actors related to governmental and non-governmental institutions, local authorities, community leaders and organized civil society to strengthen mechanisms and capacities at all levels, particularly at the community level, and achieve community empowerment that can systematically contribute to building resilience against hazards.

The proposed system allows risk assessment, prevention, and mitigation activities, as well as preparation and early warning. It involves a systematic and holistic approach to analyzing and reducing the factors that cause disasters. In this way, it is possible to prepare a national plan for comprehensive disaster risk management based on the data collected by the system at the national, regional, district, and local levels. Likewise, the goal is to reduce disaster risk so that development activities are sustainable. For this reason, the participation of all segments of society, all government components and all members of the private and professional sector is included, as suggested by the United Nations [44], ECLAC [45], and other studies [41,42,46].

That is why, through this system, communities are brought closer to technology, the digital divide is reduced, a culture of prevention is fostered, and new mechanisms of participation and motivation of the communities are created.

It is intended with this proposal to gather in a unique tool (or system) all possible information to manage disaster risk reduction, including the hospital medical system. Thus, in the event of an emergency, people with disabilities can also be accounted for, which will facilitate the management of decision makers. Therefore, the incorporation of these components into the system will create the necessary conditions for personalized health services as well as community health care.

It would be a single real-time system that can collect information from all hydrographic basins, connected and projected on maps, and from hydro-meteorological events, which are very important in the planning of the DRR. Statistical and historical data will contribute to predicting weather, defining river behavior patterns, and defining potential flood risk areas, etc. The results obtained from the operation of this system will be shared with other regions in order to help them improve or implement their projects.

## 6. Conclusions

This article describes a proposal based on the development of three research group projects in which the authors of this article participate. The three projects described are: (1) the inclusive early warning system, (2) the PwVD mobility assistance systems in public transport and indoor building environments, and (3) the dashboard for data management and decision-making. The technical solutions achieved in these projects are promising and can be integrated with each other, in different stages, to have a system on a larger scale, so that it can offer assistance in disaster risk events and other risk situations that can happen in a daily routine. There is special interest in offering aids or assistance in these matters to the visually impaired and hearing impaired, among others. The integration of the developed systems will result in a new innovative system, which is framed within the inclusive smart cities.

The results obtained to date confirm several previously stated hypotheses, for example, (a) that the proposed inclusive EWS has enabled risk assessment, prevention, and mitigation activities, as well as preparedness and early warning messages; (b) that with the development of the MOVIDIS and MOVIDIS-II projects, the community has perceived the importance of the social inclusion of visually impaired people due to the benefits that the results of these projects provide in the mobility of PwVD, in addition to the contributions that they as a community can provide; and (c) that the use of the dashboard for data management and decision-making favors the reduction of risk factors that cause disasters. Therefore, it is considered that the efficient integration of the results of these projects will make this proposal updated and interesting regarding solutions in the care and attention of visually impaired and hearing-impaired people, among others.

The inclusive early warning system for disaster risk reduction in hydrographic basins, proposed in this article, will contribute to the state of the art in this field, where the results of the three projects mentioned above are merged, including people with visual and hearing disabilities, mechanisms will be managed to have community participation, the public and private sectors will be involved, and hospital management will also be taken into account.
